# Multiple-Antenna Cooperative Spectrum Sensing Based on the Wavelet Transform and Gaussian Mixture Model

**DOI:** 10.3390/s19183863

**Published:** 2019-09-06

**Authors:** Shunchao Zhang, Yonghua Wang, Hantao Yuan, Pin Wan, Yongwei Zhang

**Affiliations:** 1 School of Automation, Guangdong University of Technology, Guangzhou 510006, China; gdut_zsc630@163.com (S.Z.); yuanhantao666@163.com (H.Y.); wanpin@gdut.edu.cn (P.W.); Yongwei_Zhang@mail2.gdut.edu.cn (Y.Z.); 2 State Key Laboratory of Management and Control for Complex Systems, Institute of Automation, Chinese Academy of Sciences, Beijing 100190, China; 3 Hubei Key Laboratory of Intelligent Wireless Communications, South-Central University for Nationalities, Wuhan 430074, China

**Keywords:** cognitive radio, spectrum sensing, multiple-antenna, wavelet transform, Gaussian mixture model

## Abstract

Spectrum sensing is a core technology in cognitive radio (CR) systems. In this paper, a multiple-antenna cooperative spectrum sensor based on the wavelet transform and Gaussian mixture model (MAWG) is proposed. Compared with traditional methods, the MAWG method avoids the derivation of the threshold and improves the performance of single secondary user (SU) spectrum sensing in cases of channel loss and hidden terminal. The MAWG method reduces the noise of the signal which collected by the multiple-antenna SUs through the wavelet transform. Then, the fusion center (FC) extracts the statistical features from the signals that are pre-processed by the wavelet transform. To extract the statistical features, an sensing data fusion method is proposed. The MAWG method divides all SUs that are involved in the cooperative spectrum sensing into two clusters and extracts a two-dimensional feature vector. In order to avoid complicated decision threshold derivation, the Gaussian mixture model (GMM) is used to train a classifier for spectrum sensing according to these two-dimensional feature vectors. Simulation experiments are performed in the κ−μ channel model. The simulation shows that the MAWG can effectively improve spectrum sensing performance under the κ−μ channel model.

## 1. Introduction

Spectrum sensing technology is used to detect and judge whether the primary user (PU) signal is present and find the spectrum holes for secondary users (SUs) to access [1,2,3]. The single SU spectrum sensing methods are susceptible to channel fadding, hidden terminal and other issues. To solve these shortcomings of classical spectrum sensing, random matrix theory (RMT) is applied to cooperative spectrum sensing (CSS), which has become a research hotspot [4]. In these methods, the covariance matrix should be calculated based on the signal matrix from SUs. Furthermore, the corresponding eigenvalue is calculated as a statistical feature of the covariance matrix for spectrum sensing. There are many CSS methods based on RMT have been proposed [5,6,7,8], such as the ratio of the maximum and minimum eigenvalue (MME), the ratio of the maximum eigenvalue to the trace (RMET), the difference between the maximum eigenvalue and the average eigenvalues (DMEAE), and the difference between the maximum and the minimum eigenvalue (DMM). These methods need to derive threshold based on the probability distribution of statistical features. However, the derivation of the threshold is usually complexity and inaccuracy.

Spectrum sensing based on multiple-antenna can also overcome the problems of traditional methods, such as noise uncertainty and path losses [9]. Multiple-antenna technology not only improves spatial diversity and spatial multiplexing gain but also effectively reduces the impact of path loss, shadows and other factors during the sensing process [10,11]. A spectrum sensing method based on the correlation coefficient by calculating the correlation coefficient between each antenna is proposed in Reference [12]. If the PU signal exists, the received signals between antennas are correlated. Otherwise the received signals between antennas are noises and have no correlation, the PU signal does not exist. According to the correlation of multiple-antenna received signals, a spectrum sensing method based on sampling covariance eigenvalue is proposed in Reference [13], which has good robustness to noise uncertainty. A CSS method for multiple-antenna CR networks based on improved energy detectors is proposed in Reference [14]. In these methods, it is also necessary to derive a decision threshold and then compare the feature of signal with the threshold to obtain a spectrum sensing decision.

The above methods all needed to derive the threshold, which affected sensing performance in some cases. Significantly, spectrum sensing schemes based on machine learning can avoid the calculation of threshold. These spectrum sensing methods are adaptive, which has been studied by many scholars. Spectrum sensing can be seen as a two-class problem in machine learning, whether the PU signal exists [15,16,17]. In [18], the energy values of the signals are used to obtain a classifier by using the K-means clustering algorithm and use the classifier to judge whether the PU exists. The maximum eigenvalue, minimum eigenvalue and the dominant eigenvalue of signals are calculated in Reference  [19]. This method combined these eigenvalues into a feature vector, and uses the K-means or Gaussian mixture model (GMM) to achieve spectrum sensing. Based on the labeled signal features, the support vector machine (SVM) and neural network (NN) in supervised learning are used to study spectrum sensing in Reference [20]. In Reference [21], a feature calculation method based on RMT is proposed, which improves the accuracy of the signal feature. K-means or K-medoids is used to train classifier for spectrum sensing. To reduce the influence of noise on spectrum sensing performance, a feature extraction method combining the empirical mode decomposition (EMD) and wavelet transform is proposed in Reference [22].

Based on the current research, to improve the spectrum sensing performance in a fading environment, this paper proposes multiple-antenna cooperative spectrum sensing based on the wavelet transform and Gaussian mixture model (MAWG), which combines the advantages of cooperative SUs, multiple-antenna and clustering algorithm. The MAWG not only reduces the influence of path losses, shadows and other factors on spectrum sensing but also avoids the complex derivation of threshold.

The main contributions of this paper can be summarized as follows.

- A new spectrum sensing method is developed to reduce the noise associated with the signal by using the wavelet transform. In this method, each SU collects spectrum sensing data from environment, performs wavelet transform to reduce noise and send the pre-processed sensing data to fusion center (FC).
- A spectrum sensing method based on GMM is proposed to avoid threshold derivation. The GMM is used to train the CSS classifier. After training, the FC uses the classifier to make the final decision about the PU state.
- In the experimental simulation section, we compare and analyze the performance of MAWG and single antenna CSS method [21,23]. These methods are simulated using the κ−μ channel. The simulation results show that the MAWG can effectively improve the spectrum sensing performance.

This paper is organized as follows. Section 2 introduces the system model of multiple-antenna CSS. Section 3 proposes a CSS method based on the GMM, which is called MAWG. Section 4 simulates the MAWG method. Results indicate that the MAWG can effectively improve the spectrum sensing performance. Section 5 summarizes the full text and outlines a simple plan for future research work.

## 2. Basic Multiple-Antenna CSS and Eigenvalues in Random Matrix

**Figure 1:**
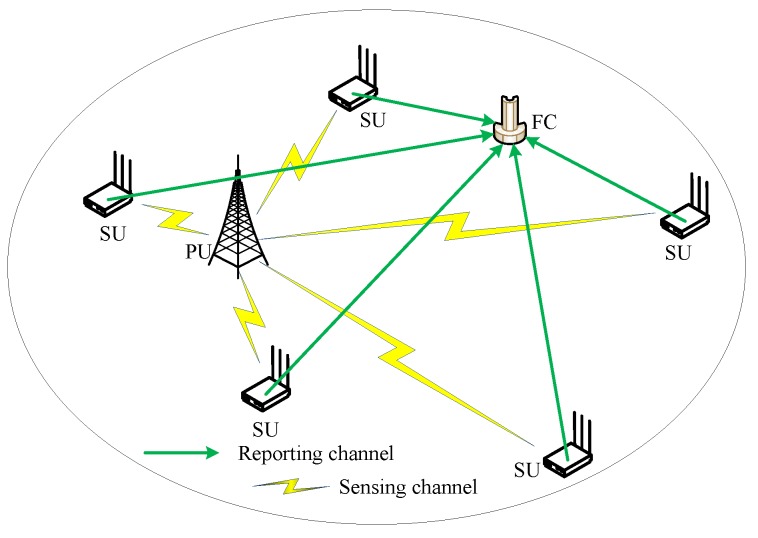
Basic multiple-antenna CSS diagram.

There are some problems in cognitive radio networks (CRN), which are path losses and shadows. It is difficult for a single SU to accurately determine and judge whether the PU is using the licensed spectrum [24,25,26,27]. Therefore, in order to combat and reduce the impact of fading channels on spectrum sensing performance, this paper study cooperative SUs with multiple-antenna for spectrum sensing. The basic multiple-antenna CSS diagram is shown in Figure 1.

According to Figure 1, it is assumed that there is only one PU, *M* cooperative SUs and a FC in the cognitive radio network. Each SU participating in the CSS has *A*antennas. The PU and the FC only have one antenna, respectively. In Figure 1, the task of each SU with multiple-antenna is collecting sensing data, using wavelet transform to reduce noise and upload the pre-processed sensing data to the FC. The FC clusters the received signals from SUs and extracts feature vectors according to different clusters. Based on these feature vectors, a classifier is trained by using GMM. After training, the classifier is used for spectrum sensing in the FC.

According to the signal status received by each antenna of the SUs, a binary hypothesis model can be expressed by [28]

(1) $$x_{i}^{l}\left(n\right)=\left\{\begin{array}{cc} w_{i}^{l}\left(n\right), & H_{0},\\ h_{i}^{l}\left(n\right)s_{i}^{l}\left(n\right)+w_{i}^{l}\left(n\right), & H_{1}, \end{array}\right.$$

n=1,2,…,N, where $x_{i}^{l}\left(n\right)$ represents the signal that is received by the *l*th antenna of the *i*th SU; $h_{i}^{l}\left(n\right)$ represents the channel gain between PU and *l*th antenna of the *i*th SU; $s_{i}^{l}\left(n\right)$ represents the signal that is transmitted by the PU; $w_{i}^{l}\left(n\right)$ represents Gaussian white noise (GWN); $H_{1}$ and $H_{0}$ represent the presence and absence of the PU signal, respectively. *N* represents the number of sampling points. In the multiple-antenna system, the received signals from different antennas exist correlation. The correlation [29] between the *a*th, a∈1,2,…,A and the *b*th, b∈1,2,…,A antenna can be defined as

(2) $$\begin{array}{c} C_{ab}=e^{-23\Lambda^{2}\left(\frac{d_{ab}}{\nu }\right)}, \end{array}$$

where $\Lambda =\frac{\sqrt{\theta^{2}+2\cos\theta -2}}{2\theta }$, $d_{ab}$ represents the distance between the *a*th and the *b*th antenna, ν represents the wavelength and θ indicates the propagation direction of antenna. If $\nu =2d_{ab}$ and θ→0 rad, the correlation $C_{ab}$ is the largest because of the $\Lambda^{2}\left(\frac{d_{ab}}{\nu }\right)\to 0$. Thus, in this condition, $s_{i}^{a}\left(n\right)=s_{i}^{b}\left(n\right)=s_{i}\left(n\right)$. In this paper, a sample condition is considered, furthermore Equations (1) is rewritten as

(3) $$x_{i}^{l}\left(n\right)=\left\{\begin{array}{cc} w_{i}^{l}\left(n\right), & H_{0},\\ h_{i}^{l}\left(n\right)s_{i}\left(n\right)+w_{i}^{l}\left(n\right), & H_{1}, \end{array}\right.$$

Based on the above assumption, the definition $x_{i}^{l}=\left[x_{i}^{l}\left(1\right),x_{i}^{l}\left(2\right),\ldots ,x_{i}^{l}\left(N\right)\right]$ represents the signal that is received by the *l*th antenna of the *i*th SU. Thus, a signal matrix can be obtained

(4) $$X_{i}=\left[\begin{array}{cccc} x_{i}^{1}\left(1\right) & x_{i}^{1}\left(2\right) & \cdots & x_{i}^{1}\left(N\right)\\ x_{i}^{2}\left(1\right) & x_{i}^{2}\left(2\right) & \cdots & x_{i}^{2}\left(N\right)\\ \vdots & \vdots & \ddots & \vdots \\ x_{i}^{A}\left(1\right) & x_{i}^{A}\left(2\right) & \cdots & x_{i}^{A}\left(N\right) \end{array}\right],$$

where $X_{i}\in R^{A\times N}$. For the convenience of the representation, the covariance matrix of the SU received signal is $R_{X_{i}}=E\left[X_{i}X_{i}^{T}\right]$. Define a signal matrix $S_{i}$ of PU received by all antennas of *i*th SU after channel losses. The covariance matrix of the $S_{i}$ is $R_{S_{i}}=E\left[S_{i}S_{i}^{T}\right]$. Further, $R_{X_{i}}$ [9,12] can be calculated by

(5) $$R_{X_{i}}=R_{S_{i}}+\sigma^{2}I,$$

where I represents the identity matrix. The eigenvalue of $R_{X_{i}}$ can be expressed by

(6) $$\lambda_{j}=\alpha_{j}+\sigma^{2},j=1,2,\ldots ,A,$$

where $\alpha_{j}$ represents the *j*th eigenvalue of $R_{S_{i}}$.

For $H_{0}$, the PU signal does not exist, and only the GWN exists in the signal matrix $X_{i}$, which means that $R_{X_{i}}=\sigma^{2}I$. At this time $\alpha_{j}=0$ and $\lambda_{max}=\lambda_{2}=\lambda_{3}=\cdots =\lambda_{min}=\sigma^{2}$.

When $H_{1}$ is established, $R_{X_{i}}=R_{S_{i}}+\sigma^{2}I$. Since the PU signal itself has a correlation, $\alpha_{j}$ makes $\lambda_{j}$ no longer be equal. Therefore, we can get $\lambda_{max}>\lambda_{2}>\lambda_{3}>\cdots >\lambda_{min}$.

In the following, the difference between the maximum eigenvalue and the minimum eigenvalue $T_{DMM}$ is calculated by

(7) $$T_{DMM}=\lambda_{max}-\lambda_{min}.$$

The ratio of the maximum eigenvalue to the matrix trace $T_{RMET}$ is calculated by

(8) $$T_{RMET}=\frac{\lambda_{max}}{\mathrm{tr}(R_{X_{i}})},$$

where tr(·) represents the trace of the matrix.

For the $T_{DMM}$ feature, when $H_{0}$ is established,

(9) $$T_{DMM}=\lambda_{max}-\lambda_{min}=0;$$

For $H_{1}$,

(10) $$T_{DMM}=\lambda_{max}-\lambda_{min}>0.$$

Equations (9) and (10) indicate that the values of $T_{DMM}$ are significantly different at $H_{0}$ and $H_{1}$, respectively. Therefore, the $T_{DMM}$ can be used for spectrum sensing.

Similarly, for the $T_{RMET}$ feature, when $H_{0}$ is established,

(11) $$T_{RMET}=\frac{\sigma^{2}}{A\sigma^{2}}=\frac{1}{A};$$

For $H_{1}$, $\lambda_{max}=\alpha_{max}+\sigma^{2}$, and

(12) $$\begin{array}{cc} T_{RMET} & =\frac{\alpha_{max}+\sigma^{2}}{\alpha_{max}+\alpha_{2}+\cdots +\alpha_{min}+A\sigma^{2}}\\ & =\frac{\alpha_{max}+\sigma^{2}}{A\bar{\alpha }+A\sigma^{2}}\\ & =\frac{1}{A}\times \frac{\alpha_{max}+\sigma^{2}}{\bar{\alpha }+\sigma^{2}}\\ & >\frac{1}{A}, \end{array}$$

where $\bar{\alpha }$ represents the average eigenvalue of $R_{S_{i}}$. We can see that the values of $T_{RMET}$ are different under the conditions of $H_{0}$ and $H_{1}$ from analyzing Equations (11) and (12), respectively. Therefore, these $T_{RMET}$ can be used for spectrum sensing.

In the experimental simulation analysis section, to effectively evaluate the performance of the MAWG algorithm, we use the detection probability $P_{d}$ and false alarm probability $P_{f}$ as the performance evaluation indicators. The specific form is as follows:

(13) $$P_{d}=P\left[{\hat{H}}_{1}|H_{1}\right],$$

where ${\hat{H}}_{1}$ is the measured status of PU being exist while $H_{1}$ is the actual status of the PU being exist.

(14) $$P_{f}=P\left[{\hat{H}}_{1}|H_{0}\right],$$

where $H_{0}$ is the actual status of the PU being absent.

For convenient reference, the symbols that are used in the paper are summarized as shown in Table 1.

## 3. Spectrum Sensing Based on GMM

The GMM is a widely used clustering algorithm which uses multiple Gaussian distributions as parameter models according to the number of clusters. The expected maximum (EM) algorithm obtains the most optimal Gaussian distribution parameters by using samples. In spectrum sensing, the presence of the PU signal and the absence of the PU signal can be considered as two different Gaussian distributions according to Equation (3). Therefore, the GMM can be used for training. It is noted that the samples are two-dimensional feature vectors extracted from the sensing signals of SUs.

### 3.1. Spectrum Sensing System Model Based on GMM

**Figure 2:**
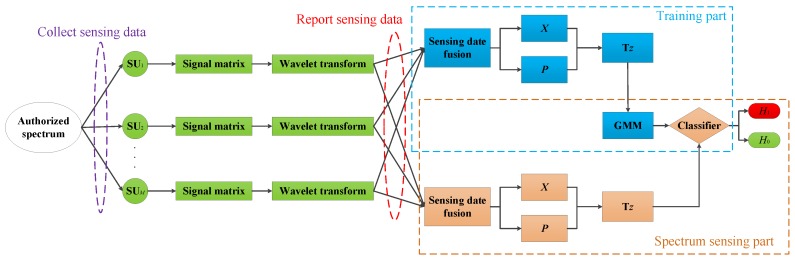
Spectrum sensing system model based on GMM.

In this section, the GMM is used for spectrum sensing. The whole process is divided into two parts, that is, the training part and the spectrum sensing part. As shown in Figure 2, the blue dotted box indicates the training part, and the yellow dotted box indicates the spectrum sensing part. Each SU previews the authorized spectrum, collects enough sensing data and pre-processes these data by Wavelet transform. Assume that these sensing data contain both states of PU. Then, the two-dimensional feature vectors $T_{z}$ are extracted from these sensing data. Finally, the classifier is trained on the FC. After the training, the classifier is used for spectrum sensing.

### 3.2. Signal Preprocessing Based on Wavelet Transform

Before calculating the two-dimensional feature vector of the SUs signals, in order to reduce the impacts of noise on the feature and improve the spectrum sesing performance under a low signal-noise ratio (SNR), the wavelet transform is used to conduct denoising in each SU. For the specific case in this paper, it is assumed that the signal collected by the *l*th antenna of the *i*th SU is $x_{i}^{l}=\left[x_{i}^{l}\left(1\right),x_{i}^{l}\left(2\right),\ldots ,x_{i}^{l}\left(N\right)\right]$ and the specific algorithm steps are as follows [30].

Step 1: The wavelet transform signal $x_{i}^{l}$ is used to obtain the wavelet coefficient *W*.

Step 2: The wavelet coefficient *W* is the threshold that is used to obtain the estimated coefficient  $\hat{W}$.

Step 3: Perform wavelet reconstruction using $\hat{W}$ and obtain denoised signal.

This paper uses a soft threshold function that is as follows:

(15) $$\hat{W}=\left\{\begin{array}{cc} sgn(W)(|W|-\beta ), & |W|\geq \beta ,\\ 0, & |W|<\beta , \end{array}\right.$$

where β is the VisuShrink threshold [31,32], which satisfies

(16) $$\beta =\sigma \sqrt{2\ln N},$$

where σ is the standard deviation of the noise.

After the signals of SUs received are reduced noise by using wavelet noise, a new signal matrix can be obtained

(17) $$J_{i}=\left[\begin{array}{cccc} y_{i}^{1}\left(1\right) & y_{i}^{1}\left(2\right) & \cdots & y_{i}^{1}\left(N\right)\\ y_{i}^{2}\left(1\right) & y_{i}^{2}\left(2\right) & \cdots & y_{i}^{2}\left(N\right)\\ \vdots & \vdots & \ddots & \vdots \\ y_{i}^{A}\left(1\right) & y_{i}^{A}\left(2\right) & \cdots & y_{i}^{A}\left(N\right) \end{array}\right].$$

After sensing data is pre-processed by SUs, these sensing data is uploaded to the FC. In the CSS, i>2. To extract signal feature, a sensing data fusion method is used to obtain a two-dimensional feature vector. It is noted that the fusion method can fuse the sensing data from SUs which equip different number antennas. Specifically, The FC divides the SUs into two clusters $C_{1}$ and $C_{2}$. When i≥2 and *M* is an odd number, let $J_{1},J_{3},\ldots ,J_{M}\in C_{1}$ and $J_{2},J_{4},\ldots ,J_{M-1}\in C_{2}$. When i≥2 and *M* is even, let $J_{1},J_{3},\ldots ,J_{M-1}\in C_{1}$ and $J_{2},J_{4},\ldots ,J_{M}\in C_{2}$. Then, the matrices in $C_{1}$ and $C_{2}$ are recombined to obtain matrices X and P.

When i≥2 and *M* is an odd number, the signal data that is collected by the SUs in the recombination cluster $C_{1}$ can obtain X, which is a $\frac{(M+1)A}{2}\times N$ matrix

(18) $$X=\left[\begin{array}{cccc} y_{1}^{1}\left(1\right) & y_{1}^{1}\left(2\right) & \cdots & y_{1}^{1}\left(N\right)\\ y_{1}^{2}\left(1\right) & y_{1}^{2}\left(2\right) & \cdots & y_{1}^{2}\left(N\right)\\ \vdots & \vdots & \ddots & \vdots \\ y_{1}^{A}\left(1\right) & y_{1}^{A}\left(2\right) & \cdots & y_{1}^{A}\left(N\right)\\ \vdots & \vdots & \ddots & \vdots \\ y_{i}^{l}\left(1\right) & y_{i}^{l}\left(2\right) & \cdots & y_{i}^{l}\left(N\right)\\ \vdots & \vdots & \ddots & \vdots \\ y_{M}^{1}\left(1\right) & y_{M}^{1}\left(2\right) & \cdots & y_{M}^{1}\left(N\right)\\ y_{M}^{2}\left(1\right) & y_{M}^{2}\left(2\right) & \cdots & y_{M}^{2}\left(N\right)\\ \vdots & \vdots & \ddots & \vdots \\ y_{M}^{A}\left(1\right) & y_{M}^{A}\left(2\right) & \cdots & y_{M}^{A}\left(N\right) \end{array}\right].$$

By reorganizing the matrix in cluster $C_{2}$, P can be obtained as a $\frac{(M-1)A}{2}\times N$ matrix

(19) $$P=\left[\begin{array}{cccc} y_{2}^{1}\left(1\right) & y_{2}^{1}\left(2\right) & \cdots & y_{2}^{1}\left(N\right)\\ y_{2}^{2}\left(1\right) & y_{2}^{2}\left(2\right) & \cdots & y_{2}^{2}\left(N\right)\\ \vdots & \vdots & \ddots & \vdots \\ y_{2}^{A}\left(1\right) & y_{2}^{A}\left(2\right) & \cdots & y_{2}^{A}\left(N\right)\\ \vdots & \vdots & \ddots & \vdots \\ y_{i}^{l}\left(1\right) & y_{i}^{l}\left(2\right) & \cdots & y_{i}^{l}\left(N\right)\\ \vdots & \vdots & \ddots & \vdots \\ y_{M-1}^{1}\left(1\right) & y_{M-1}^{1}\left(2\right) & \cdots & y_{M-1}^{1}\left(N\right)\\ y_{M-1}^{2}\left(1\right) & y_{M-1}^{2}\left(2\right) & \cdots & y_{M-1}^{2}\left(N\right)\\ \vdots & \vdots & \ddots & \vdots \\ y_{M-1}^{A}\left(1\right) & y_{M-1}^{A}\left(2\right) & \cdots & y_{M-1}^{A}\left(N\right) \end{array}\right].$$

Similarly, when i≥2 and *M* is even, X and P are also obtained, and both are $\frac{MA}{2}\times N$ matrices.

According to the obtained X and P matrices, the corresponding covariance matrices $R_{X}=E\left[XX^{T}\right]$ and $R_{P}=E\left[PP^{T}\right]$ are respectively calculated.

When i≥2 and *M* is an odd number, it is assumed that the eigenvalues of matrices $R_{X}$ and $R_{P}$ are $\lambda_{1}\left(\lambda_{max}\right)>\lambda_{2}>\lambda_{3}>\cdots >\lambda_{(M+1)A/2}\left(\lambda_{min}\right)\;\mathrm{or}\;\lambda_{(M-1)A)/2}\left(\lambda_{min}\right)$ from the maximum to the minimum. When i≥2 and *M* is even, then the eigenvalues of matrices $R_{X}$ and $R_{P}$ are $\lambda_{1}\left(\lambda_{max}\right)>\lambda_{2}>\lambda_{3}>\cdots >\lambda_{MA/2}\left(\lambda_{min}\right)$ from the maximum to the minimum. According to this, $T_{DMM}$ can be obtained by

(20) $$T_{DMM}=\lambda_{max}-\lambda_{min}.$$

$T_{RMET}$ can be calculated by

(21) $$T_{RMET}=\lambda_{max}/\mathrm{tr}\left(R_{g}\right).\;g\in \left\{X,P\right\}$$

Based on the covariance matrix, the corresponding statistical feature $T_{DMM}$ or $T_{RMET}$ is calculated. Let $T_{R_{X},z}$ and $T_{R_{P},z}$, where z∈{DMM,RMET}, denote the features corresponding to $R_{X}$ and $R_{P}$, respectively. Thus, a feature vector is obtained

(22) $$T_{z}={\left[T_{R_{X},z}\;T_{R_{P},z}\right]}^{T},\;z\in \left\{DMM,RMET\right\}.$$

In the next section, the classifier is trained by using a sufficient number of $T_{z}$ feature vectors and GMM, which is used to achieve spectrum sensing.

### 3.3. Offline Training Based on GMM

Before training begins, we need to prepare a training feature vectors set [33],

(23) $$S=\{T_{z}^{1},T_{z}^{2},\ldots ,T_{z}^{B}\},$$

where *B* is the number of training feature vectors, $T_{z}^{b},b=1,2,\ldots ,B$ is the *b*th feature vector that is extracted according to the method that is proposed in this paper. The distributions of GMM can be expressed by [19]

(24) $$p\left(x\right)=\sum_{k}^{K}\pi_{k}N\left(x|\mu_{k},\Sigma_{k}\right).$$

where *K* represents the number of mixed components, $\pi_{k}$ is the mixing coefficient that satisfies $\sum_{k}^{K}\pi_{k}=1$, $N(x|\mu_{k},\Sigma_{k})$ is a Gaussian distribution with a mean of $\mu_{k}$ and a variance of $\Sigma_{k}$,

(25) $$N\left(x|\mu_{k},\Sigma_{k}\right)=\frac{1}{{\left(2\pi \right)}^{\frac{D}{2}}{\left|\Sigma_{k}\right|}^{\frac{1}{2}}}e^{-\frac{1}{2}{\left(x-\mu_{k}\right)}^{T}\Sigma_{k}^{-1}\left(x-\mu_{k}\right)}.$$

According to the situation of spectrum sensing, spectrum sensing can be considered as a two-class problem whether the PU is using the licensed spectrum, which means K=2. Thus, Equation (24) can be rewritten as

(26) $$p\left(x\right)=\pi_{1}N\left(x|\mu_{1},\Sigma_{1}\right)+\pi_{2}N\left(x|\mu_{2},\Sigma_{2}\right).$$

The maximum likelihood function is as follows:

(27) $$\ln p\left(S|\pi ,\mu ,\Sigma \right)=\sum_{b=1}^{B}\ln\left\{\sum_{b=1}^{K}\pi_{k}N\left(T_{z}^{b}|\mu_{k},\Sigma_{k}\right)\right\}.$$

To solve the parameters in Equation (26), the maximum likelihood function Equation (27) is used to estimate the parameters $\left(\pi_{1},\mu_{1},\Sigma_{1}\right)$ and $\left(\pi_{2},\mu_{2},\Sigma_{2}\right)$.

The process is described by Algorithm 1.

**Algorithm 1**: Offline training based on GMM.**Initialization**: K=2, $\pi_{k}$, $\mu_{k}$, $\Sigma_{k}$, where k=1,2.**Repeat**:**Step 1**: Calculate the posterior probability γ(b,k) according to the current $\pi_{k}$, $\mu_{k}$, $\Sigma_{k}$.           $\gamma \left(b,k\right)=\frac{\pi_{k}N\left(T_{z}^{b}|\mu_{k},\Sigma_{k}\right)}{\sum_{j=1}^{K}\pi_{j}N\left(T_{z}^{b}|\mu_{j},\Sigma_{j}\right)}$, where k=1,2 and b=1,2,…,B.**Step 2**: According to γ(b,k), calculate $\pi_{k}$, $\mu_{k}$, $\Sigma_{k}$.           $\mu_{k}=\frac{1}{B_{k}}\sum_{b=1}^{B}\gamma \left(b,k\right)T_{z}^{b}$,           $\Sigma_{k}=\frac{1}{B_{k}}\sum_{b=1}^{B}\gamma \left(b,k\right)\left(T_{z}^{b}-\mu_{k}\right){\left(T_{z}^{b}-\mu_{k}\right)}^{T}$,           $\pi_{k}=\frac{B_{k}}{B}$,           where $B_{k}=\sum_{b=1}^{B}\gamma \left(b,k\right)$.**Step 3**: Check whether the parameters converge. If they do not converge, return to **Step 1** and continue           executing the algorithm.
**End**


### 3.4. Online Spectrum Sensing Based on GMM

After the training is completed, the optimal parameters $\pi_{k}^{*}$, $\mu_{k}^{*}$, and $\Sigma_{k}^{*}$ can be obtained. According to these optimal parameters, a classifier for spectrum sensing can be constructed

(28) $$\ln\frac{\pi_{1}^{*}N\left(T_{z}|\mu_{1}^{*},\Sigma_{1}^{*}\right)}{\pi_{2}^{*}N\left(T_{z}|\mu_{2}^{*},\Sigma_{2}^{*}\right)}>\xi .$$

where, the parameter ξ is used to control $P_{f}$ in the spectrum sensing system. If ξ is smaller, the PU is more likely not to use the authorized channel, which means the channel is available. Then, the probability of miss detection and $P_{f}$ are increased. Conversely, if the ξ is larger, the PU is more likely to use the authorized channel, which means the channel is unavailable. Hence, the $P_{d}$ and spectrum utilization are reduced.

When performing online sensing, the two-dimensional feature vector ${\bar{T}}_{z}$ are extracted from the channel which needs to be perceived. Finally, we use Equation (28) for spectrum sensing.

## 4. Experimental Simulation Analysis

In this section, the MAWG method is verified in κ−μ channel fading model. The κ−μ channel model is a widely accepted model because it can generate many known wireless channel models by adjusting the parameters κ and μ. By setting κ and μ in the κ−μ fading channel to some specific parameter values, it can be converted into known models, such as the Rayleigh fading channel (μ=1,κ→0), the Rician channel (μ=1) and the Nakagami-*m* channel (κ→0).

To demonstrate the performance of the MAWG method, in the simulation experiment, DMM or RMET is selected as the characteristic of the signal. The PU signal is a multiple component signal [22] in this experiment. According to the spectrum sensing statistical feature extraction method that is described above, 2000 feature vectors are extracted. Firstly, 1000 feature vectors are used to train the GMM framework. After the training is completed, the classifiers which are used for spectrum sensing are obtained. Then, the other 1000 feature vectors are used for testing.

### 4.1. Clustering Performance Analysis

**Figure 3:**
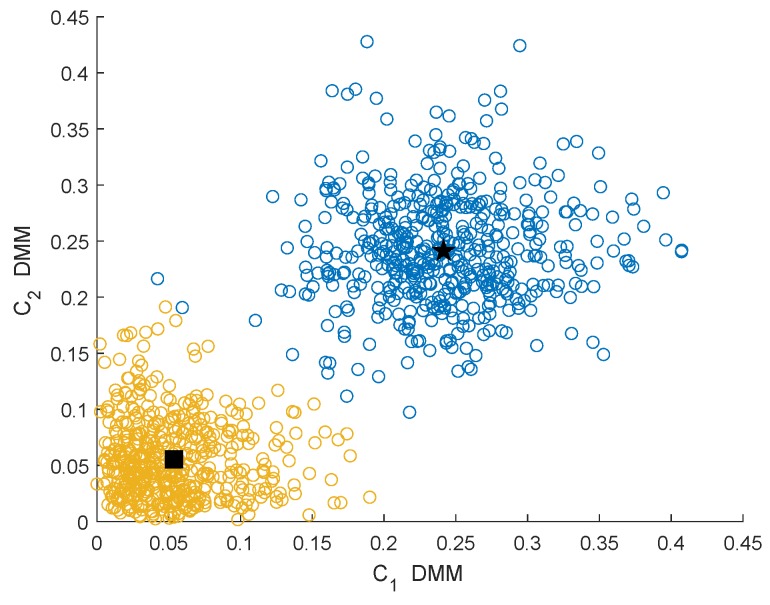
Clustering effect of the differences between maximum and minimum eigenvalue (DMMs) under the Rayleigh channel.

**Figure 4:**
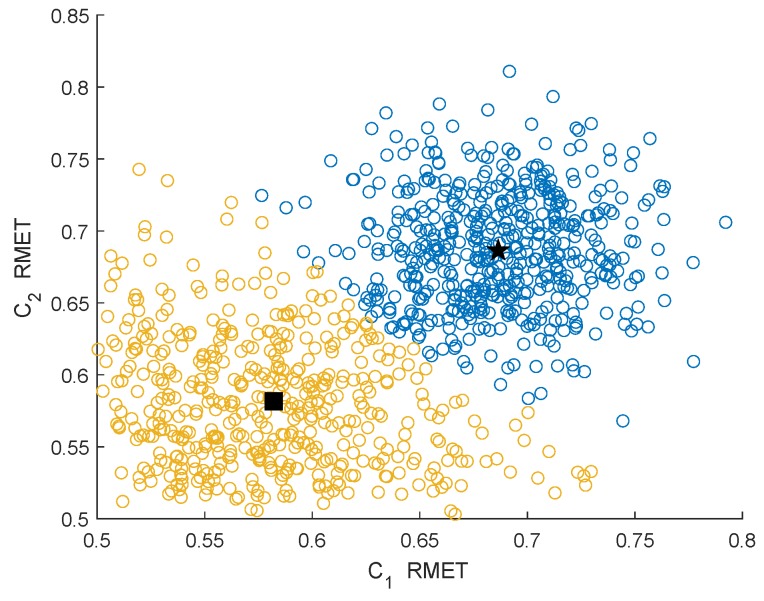
Clustering effect of the ratios of the maximum eigenvalue to the trace (RMETs) under the Rayleigh channel.

In this section, we analyze the clustering effect of the GMM clustering algorithm under different characteristics and different channel conditions. The channel conditions are the Rayleigh fading channel (μ=3,κ→0) and Rician fading channel (μ=1,κ=3). Figure 3 and Figure 4 show the clustering effects of the different features under Rayleigh channel with an SNR =−10 dB.

**Figure 5:**
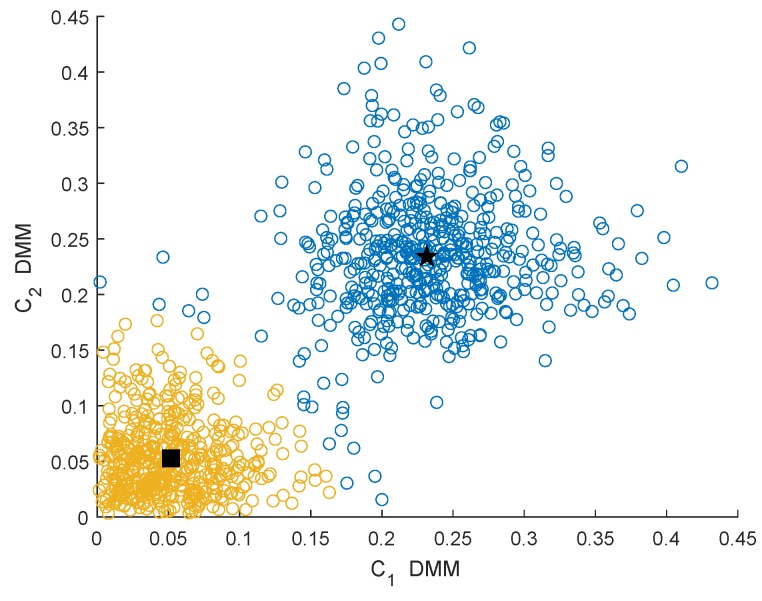
Clustering effect of the DMMs under the Rician channel.

**Figure 6:**
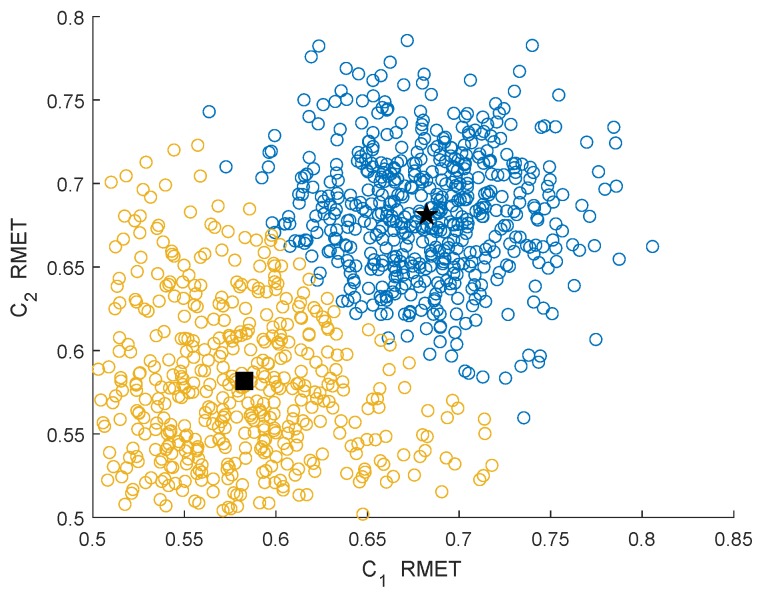
Clustering effect of the RMETs under the Rician channel.

Figure 5 and Figure 6 show the clustering effects of the different features under the Rician channel with a SNR =−10 dB.

In Figure 3, Figure 4, Figure 5 and Figure 6, the yellow circles represent the feature vectors that are classified as noise. The blue circles represent the feature vectors that are classified as the PU signal existence class. The star represents the mean $\mu_{2}^{*}$ of the PU signal existence classes. The square represents the mean $\mu_{1}^{*}$ of the noise class.

By observing and analyzing Figure 3, Figure 4, Figure 5 and Figure 6, it can be found that the DMM feature can contain higher characteristic information for the reaction signal. Therefore, in the following simulation analysis, the simulation experiments are mainly carried out for DMM features in the MAWG method, which is called the MAWGDMM method.

### 4.2. Experimental Results and Performance Analysis with Different SNR

**Figure 7:**
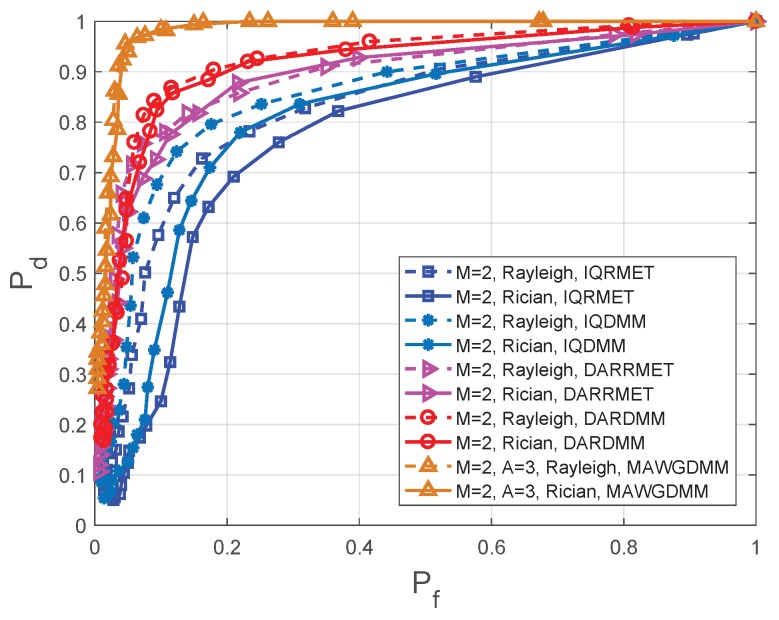
Comparison of the receiver operating characteristics (ROC) in different methods at SNR =−14 dB.

**Figure 8:**
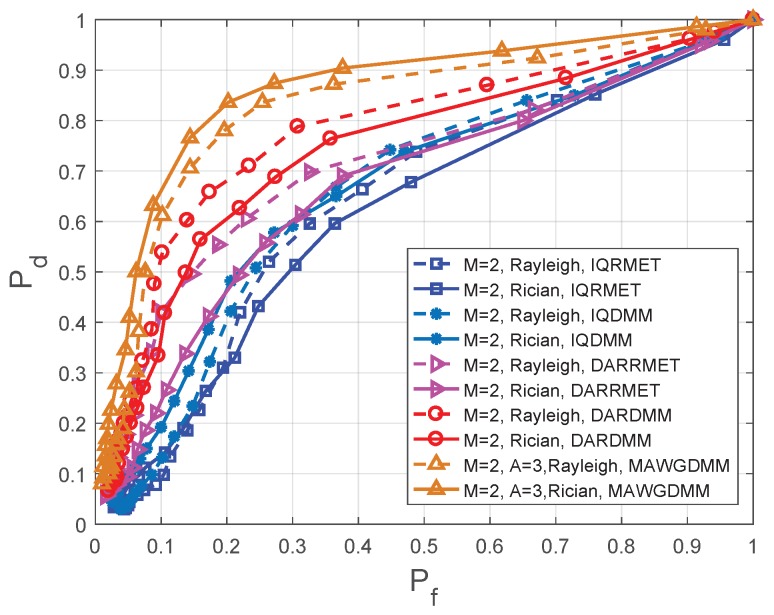
Comparison of ROC in different methods at SNR =−16 dB.

The IQDMM and IQRMET methods in Figure 7 and Figure 8 are proposed in Reference [21]. DARDMM and DARRMET are proposed in Reference [23]. These methods use the IQ and DAR decomposition to increase the logic SUs. In the feature extraction, the DMM and RMET were chosen to construct feature vector. For achieve CSS, the K-means is used in [21] and K-medoids is used in Reference [23]. The simulation parameters are set as follows in the MAWGDMM algorithm: the number of SUs is M=2, the number of sampling points is N=1000 and the number of antennas is A=3.

Figure 7 shows the simulation results when the SNR =−14 dB with Rayleigh and Rician fading channels. Figure 8 shows the simulation results when the SNR =−16 dB with Rayleigh and Rician fading channels. Table 2 and Table 3 show the detection probabilities of the different algorithms under different fading channels at the same false alarm probability. The receiver operating characteristics (ROC) have been drawn for a comparative performance analysis.

Multiple-antenna spectrum sensing can make full use of multiplexing and spatial diversity, which can reduces the effects of path losses and shadows on spectrum sensing. Thus, according to Figure 7 and Figure 8, Table 2 and Table 3, it can conclude that the MAWGDMM has better spectrum sensing.

When the SNR =−14 dB, $P_{f}=0.1$, a Rayleigh channel is used. The spectrum sensing performance of the MAWGDMM algorithm relative to DARDMM, DARRMET, IQDMM, and IQRMET algorithms is increased by 15.29%, 25.64%, 42.03% and 66.10%, respectively. When $P_{f}=0.2$, the spectrum sensing performance is improved by 9.89%, 17.65%, 33.33%, and 49.25%, respectively. When using the Rician channel and $P_{f}=0.1$, the performance is improved by 17.86%, 33.78%, 141.46%, and 312.50%, respectively. The performance is improved by 11.11%, 16.28%, 23.46%, and 33.33%, respectively, when $P_{f}=0.2$.

In SNR =−16 dB, the sensing performance of the MAWGDMM algorithm is analysed in the Rayleigh channel and the Rician channel, respectively. From the first row in Table 3, when using Rayleigh and $p_{f}=0.1$, the performance of the MAWGDMM method is 15.09%, 45.24%, 369.23% and 510.00% higher than DARDMM, DARRMET, IQDMM and IQRMET, respectively. When using Rayleigh and $P_{f}=0.2$, the sensing performance of MAWGDMM methd is improved by 16.42%, 36.84%, 95.00% and 122.86%, respectively. Under the Rician channel, the performance is improved by 80.56%, 170.83%, 242.11%, and 364.29%, respectively, when $P_{f}=0.1$. The performance is improved by 37.70%, 82.61%, 82.61%, and 162.50%, respectively, when $P_{f}=0.2$.

### 4.3. Performance Analysis with Different Values of A and M

**Figure 9:**
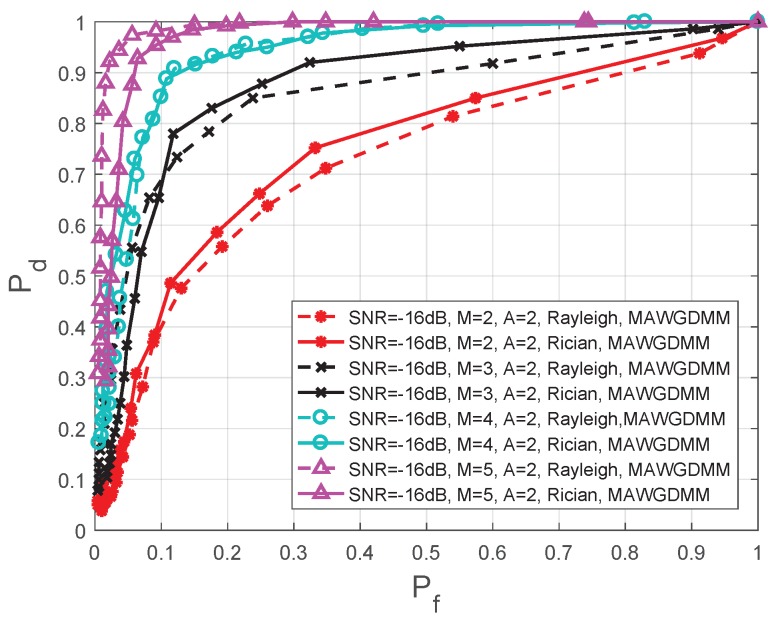
Comparison of ROC in different number of SUs.

This section analyzes the impacts of different number of cooperative SUs and the number of different antennas on the spectrum sensing performance. In Figure 9, the simulation parameters are set as follows—SNR =−16 dB, N=1000, A=2, and *M* is 2, 3, 4, 5, respectively.

As the number of SUs increases, more comprehensive information for the PU signal is collected, which can overcome the problem hidden terminal and improve the spatial diversity. The final decision of the FC is more reliable. Thus, from Figure 9 and Table 4, we can see that the spectrum sensing performance is further improved as the number of SUs increases.

When the SNR =−16 dB, a Rayleigh channel is used, and $P_{f}=0.1$; the spectrum perceptual performance when M=5 is improved by 15.29%, 44.12%, and 145.00%, respectively, compares to conditions when M=4, M=3, and M=2. When $P_{f}=0.2$, the perceived performance is increased by 5.32%, 22.22%, and 47.76%, respectively. Under a Rician channel, the performance is increased by 12.94%, 41.18%, and 134.15%, respectively, when $P_{f}=0.1$; the performance is increased by 4.26%, 16.67%, and 36.11%, respectively, when $P_{f}=0.2$.

**Figure 10:**
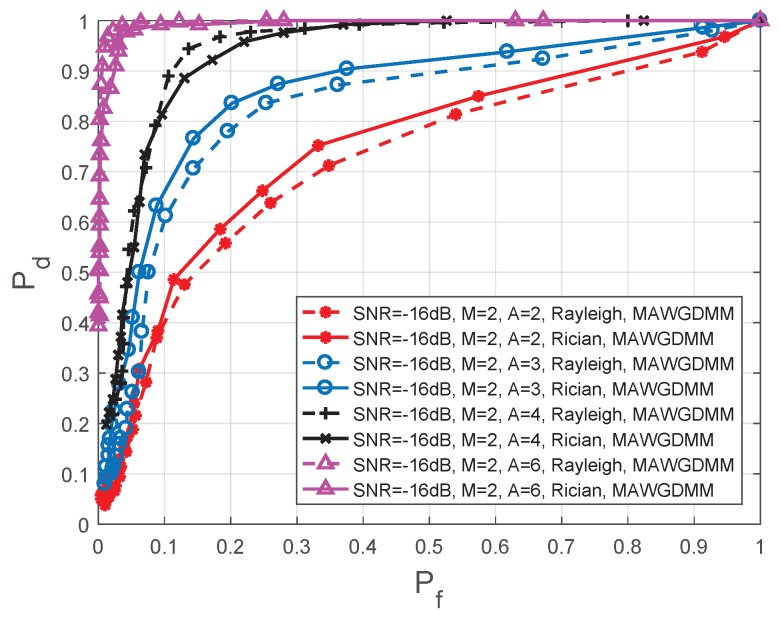
Comparison of ROC in different number of antennas.

In Figure 10, the simulation parameters are set as follows in the MAWGDMM algorithm: SNR =−16 dB, M=2, N=1000, and *A* is 2, 3, 4, and 6, respectively.

As the number of SU antennas increases, the spatial diversity and spatial multiplexing gain are improved. The MAWGDMM can work well when many antennas observe the authorized spectrum together. By analyzing Figure 10 and Table 5, we can conclude that the spectrum sensing performance is improved.

When the SNR =−16 dB, a Rayleigh channel is used and $P_{f}=0.1$, the spectrum sensing performance when A=6 is improved by 22.22%, 65.00%, and 153.85%, respectively, compares to conditions when A=4, A=3, and A=2. When $P_{f}=0.2$, the performance is increased by 2.04%, 26.59%, and 75.44%, respectively. Under a Rician channel, the performance is increased by 15.29%, 48.48%, and 145.00% respectively, when $P_{f}=0.1$, and the performance is increased by 4.21%, 19.28%, and 65.00%, respectively, when $P_{f}=0.2$.

### 4.4. Performance Analysis with Different Numbers of Sample Points

**Figure 11:**
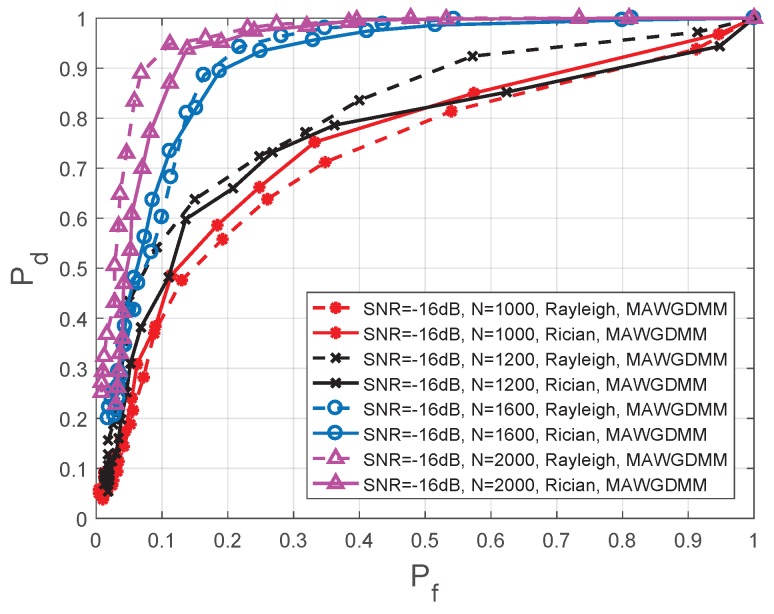
Comparison of ROC in different number of sampling points.

This section will analyze the effect of different numbers of samples on the spectrum sensing performance. In Figure 11, the simulation parameters are set as follows—the SNR =−16 dB, M=2, A=2, *N* is 1000, 1200, 1600, and 2000, respectively.

As the number of sampling points increases, a more complete PU signal can be collected by the SUs. The feature vector is more representative of the status of the PU. By analyzing Figure 11 and Table 6, it can be concluded that as the number of sample points increases, the spectrum sensing performance is improved.

When the SNR=−16 dB, a Rayleigh channel is used and $P_{f}=0.1$; the spectrum sensing performance when N=2000 is improved by 56.67%, 70.9%, and 135.00%, respectively, compares to conditions when N=1600, N=1200, and N=1000. In the case when $P_{f}=0.2$, the performance is increased by 4.35%, 41.18%, and 68.42%, respectively. Under a Rician channel, the performance is increased by 17.14%, 78.26%, and 100.00%, respectively, when $P_{f}=0.1$, and the performance is increased by 6.67%, 45.45%, and 60.00%, respectively, when $P_{f}=0.2$.

## 5. Conclusions

This paper aims to improve spectrum sensing performance, especially the spectrum sensing performance of the fading channel. Based on this aim, this paper proposes the multiple-antenna CSS based on the wavelet transform and GMM. This method adopts cooperative SUs and the multiple antenna spectrum sensing method, which can effectively overcome the problems that are encountered by single SU spectrum sensing, such as path losses and shadows. Specifically, this paper proposes a new signal feature extraction method and combines the GMM to achieve spectrum sensing. In the experimental simulation section, the simulation with the κ−μ channel is performed and the simulation results are analyzed. The results show that the MAWG method can improve the spectrum sensing performance to some extent. In this paper, the analysis of the overall cost is ignored; in future research, we will further analyze the overall cost and improve the applicability of the algorithm.

## Figures and Tables

**Table 1:** Symbols and notations.

Symbol	Notations
$w_{i}^{l}\left(n\right)$	Noise signal received by the *l*th antenna of *i*th SU at time *n*
s(n)	PU signal at time *n*
$h_{i}^{l}\left(n\right)$	Channel loss between PU and the *l*th antenna of *i*th SU at time *n*
$x_{i}^{l}\left(n\right)$	The signal received by the *l*th antenna of the *i*th SU at time *n*
*N*	Sampling points
$H_{1},H_{0}$	PU signal exists, PU does not exists
${\hat{H}}_{1},$	The measured status of PU being exist
*M*	Number of SUs
*A*	Number of antennas
$X_{i}$	Signal matrix collected by *i*th SU
$S_{i}$	PU signal matrix collected by *i*th SU
$R_{X_{i}}$, $R_{S_{i}}$	Covariance matrix of $X_{i}$ and $S_{i}$
*I*	Identity matrix
$\lambda_{j}$	The *j*th eigenvalue of $R_{X_{i}}$
$\alpha_{j}$	The *j*th eigenvalue of $R_{S_{i}}$
$\bar{\alpha }$	Average value of $\alpha_{j}$, where j=1,2,…,A
$P_{f},P_{d}$	False alarm probability, detection probability
*W*	Wavelet coefficient
$\hat{W}$	Estimated coefficient
β	VisuShrink threshold
σ	Standard deviation of noise
$J_{i}$	$X_{i}$ after wavelet transform
$C_{1}$, $C_{2}$	Two clusters composed of different SUs
*X*, *P*	Matrix corresponding to $C_{1}$ and $C_{2}$
$R_{X}$, $R_{P}$	Covariance matrix of to *X* and *P*
$T_{DMM}$	DMM eigenvalue
$T_{RMET}$	RMET eigenvalue
$T_{X,z}$, $T_{P,z}$	Feature from $R_{X}$ and $R_{P}$.
$T_{z}$	A feature vector composed of $T_{X,z}$ and $T_{P,z}$, where z∈{DMME,RMET}
*S*	Training feature set
$T_{z}^{b}$	The *b*th training feature $T_{z}$
p(x)	Gaussian distribution
$\pi_{k}$	Mixing coefficien
$\Sigma_{k}$	Variance
$\mu_{k}$	Mean
*K*	Number of clusters
κ−μ	Channel loss model
ξ	Threshold for controlling $P_{f}$ and $P_{d}$

**Table 2:** Detection probabilities of different algorithms when the SNR =−14 dB.

	MAWGDMM	DARDMM	DARRMET	IQDMM	IQRMET
Rayleigh, $P_{f}=0.1$	0.98	0.85	0.78	0.69	0.59
Rayleigh, $P_{f}=0.2$	1.00	0.91	0.85	0.75	0.67
Rician, $P_{f}=0.1$	0.99	0.84	0.74	0.41	0.24
Rician, $P_{f}=0.2$	1.00	0.90	0.86	0.81	0.75

**Table 3:** Detection probabilities of different algorithms when the SNR =−16 dB.

	MAWGDMM	DARDMM	DARRMET	IQDMM	IQRMET
Rayleigh, $P_{f}=0.1$	0.61	0.53	0.42	0.13	0.10
Rayleigh, $P_{f}=0.2$	0.78	0.67	0.57	0.40	0.35
Rician, $P_{f}=0.1$	0.65	0.36	0.24	0.19	0.14
Rician, $P_{f}=0.2$	0.84	0.61	0.46	0.46	0.32

**Table 4:** Detection probabilities of different numbers of secondary users (SUs) when the SNR =−16 dB.

	M=2	M=3	M=4	M=5
Rayleigh, $P_{f}=0.1$	0.40	0.68	0.85	0.98
Rayleigh, $P_{f}=0.2$	0.67	0.81	0.94	0.99
Rician, $P_{f}=0.1$	0.41	0.68	0.85	0.96
Rician, $P_{f}=0.2$	0.72	0.84	0.94	0.98

**Table 5:** Detection probabilities for the different numbers of antennas when the SNR =−16 dB.

	A=2	A=3	A=4	A=6
Rayleigh, $P_{f}=0.1$	0.39	0.60	0.81	0.99
Rayleigh, $P_{f}=0.2$	0.57	0.79	0.98	1.00
Rician, $P_{f}=0.1$	0.40	0.66	0.85	0.98
Rician, $P_{f}=0.2$	0.60	0.83	0.95	0.99

**Table 6:** Detection probabilities of different numbers of sampling points when the SNR =−16 dB.

	N=1000	N=1200	N=1600	N=2000
Rayleigh, $P_{f}=0.1$	0.40	0.55	0.60	0.94
Rayleigh, $P_{f}=0.2$	0.57	0.68	0.92	0.96
Rician, $P_{f}=0.1$	0.41	0.46	0.70	0.82
Rician, $P_{f}=0.2$	0.60	0.66	0.90	0.96

## Abbreviations

The following abbreviations are used in this manuscript:

CR	Cognitive radio
CRN	Cognitive radio networks
MAWG	A multiple-antenna cooperative spectrum sensing based on the wavelet transform and Gaussian
	mixture model
GMM	Gaussian mixture model
SU	Secondary user
FC	Fusion center
PU	Primary user
RMT	Random matrix theory
CSS	Cooperative spectrum sensing
MME	The ratio of the maximum and minimum eigenvalue
DMEAE	The maximum eigenvalue and the average eigenvalues
DMM	the difference between the maximum and the minimum eigenvalue
SVM	Support vector machine
NN	Neural network
EMD	Empirical mode decomposition
GWN	Gaussian white noise
SNR	Signal-noise ratio
ROC	Receiver operating characteristics
